# Indoxyl Sulfate Contributes to Adipose Tissue Inflammation through the Activation of NADPH Oxidase

**DOI:** 10.3390/toxins12080502

**Published:** 2020-08-05

**Authors:** Shoma Tanaka, Hiroshi Watanabe, Takehiro Nakano, Tadashi Imafuku, Hiromasa Kato, Kai Tokumaru, Nanaka Arimura, Yuki Enoki, Hitoshi Maeda, Motoko Tanaka, Kazutaka Matsushita, Masafumi Fukagawa, Toru Maruyama

**Affiliations:** 1 Department of Biopharmaceutics, Graduate School of Pharmaceutical Sciences, Kumamoto University, Kumamoto 8620973, Japan; muys102@gmail.com (S.T.); 152p1032@st.kumamoto-u.ac.jp (T.N.); ttt.iiii.0514@gmail.com (T.I.); 181y3001@st.kumamoto-u.ac.jp (H.K.); 161p1034@st.kumamoto-u.ac.jp (K.T.); 174p1003@st.kumamoto-u.ac.jp (N.A.); maeda-h@kumamoto-u.ac.jp (H.M.); 2 Division of Pharmacodynamics, Keio University Faculty of Pharmacy, Tokyo 1058512, Japan; enoki-yk@pha.keio.ac.jp; 3 Department of Nephrology, Akebono Clinic, Kumamoto 8614112, Japan; tanaka@matusita-kai.or.jp (M.T.); kmatu_ipadc@i.softbank.jp (K.M.); 4 Division of Nephrology, Endocrinology and Metabolism, Tokai University School of Medicine, Kanagawa 2591193, Japan; fukagawa@tokai-u.jp

**Keywords:** indoxyl sulfate, adipocyte, chronic inflammation, AST-120, NADPH oxidase, reactive oxygen species

## Abstract

Adipose tissue inflammation appears to be a risk factor for the progression of chronic kidney disease (CKD), but the effect of CKD on adipose tissue inflammation is poorly understood. The purpose of this study was to clarify the involvement of uremic toxins (indoxyl sulfate (IS), 3-indoleacetic acid, p-cresyl sulfate and kynurenic acid) on CKD-induced adipose tissue inflammation. IS induces monocyte chemoattractant protein-1 (MCP-1) expression and reactive oxygen species (ROS) production in the differentiated 3T3L-1 adipocyte. An organic anion transporter (OAT) inhibitor, an NADPH oxidase inhibitor or an antioxidant suppresses the IS-induced MCP-1 expression and ROS production, suggesting the OAT/NADPH oxidase/ROS pathway is involved in the action of IS. Co-culturing 3T3L-1 adipocytes and mouse macrophage cells showed incubating adipocytes with IS increased macrophage infiltration. An IS-overload in healthy mice increased IS levels, oxidative stress and MCP-1 expression in epididymal adipose tissue compared to unloaded mice. Using 5/6-nephrectomized mice, the administration of AST-120 suppressed oxidative stress and the expression of MCP-1, F4/80 and TNF-α in epididymal adipose tissue. These collective data suggest IS could be a therapeutic target for the CKD-related inflammatory response in adipose tissue, and that AST-120 could be useful for the treatment of IS-induced adipose tissue inflammation.

## 1. Introduction

Chronic kidney disease (CKD), a chronic inflammatory disease, involves oxidative stress and inflammation. In recent years, adipose tissue inflammation has attracted attention as a risk factor for the progression of CKD and the subsequent development of its complications. In fact, a meta-analysis of clinical trials showed that obesity was independently associated with reduced estimated glomerular filtration rate (eGFR) and albuminuria [1,2]. In addition, it has been reported that changes in adipose tissue volume are correlated with the plasma concentration of C-reactive protein, a marker of inflammation, and of the levels of active sCD163, a marker of activated macrophages. These findings suggest that adipose tissue-derived inflammation could be a factor in the development of renal damage that is associated with obesity. On the other hand, it was also reported that adipose tissue inflammation is accompanied by macrophage infiltration in subcutaneous fat and visceral fat, even in CKD patients who are not obese [3,4,5]. These data indicate that CKD and obesity share similar pathological features in adipose tissue. Such adipose tissue inflammation is thought to be involved in the further exacerbation of oxidative stress and inflammation as observed in CKD [6]. Although adipose tissue inflammation is generally considered to be associated with chronic inflammation in CKD, there are still a number of unclear points regarding the factor that induces adipose tissue inflammation and its molecular mechanism in cases of CKD.

Uremic toxins are substances that accumulate in the body due to decreased renal function, and it has been reported that they are involved in damage to distant organs as a humoral factor. We recently reported that, among the anionic protein-binding uremic substances, indoxyl sulfate contributes to sarcopenia in CKD by inducing muscle atrophy and muscular mitochondrial damage [7,8,9]. Moreover, we also reported that p-cresyl sulfate is involved in cardiovascular disorders that are associated with vascular endothelial cell injury and vascular smooth muscle calcification [10], in addition to the progression of renal injury/renal fibrosis [11]. In addition, we demonstrated that oral charcoal adsorbent AST-120 administration, which reduced plasma concentration of these uremic toxins, to CKD mice ameliorated the progression of CKD, CKD-induced sarcopenia and vascular injury [10,11]. As a mechanism of cytotoxicity caused by such uremic toxins, we found that cellular uptake via organic anion transporter (OAT), followed by an increase in the levels of reactive oxygen species (ROS) through NADPH oxidase activation [12,13] and an increase in inflammatory cytokine levels are involved in this process. In general, chronic oxidative stress exposure enhances the production of the chemokine monocyte chemoattractant protein-1 (MCP-1) in adipose tissue. MCP-1 induces the infiltration of macrophages into adipose tissue via the C-C chemokine receptor-2 (CCR2), a receptor that is expressed on macrophages. Adipocytes then cause an increase in the production of inflammatory adipocytokines, such as TNF-α, and induce inflammatory changes [14]. Based on these data, we hypothesize that uremic toxins may also induce inflammation by increasing ROS production, even in adipose tissue, and AST-120 may reduce the uremic toxins-induced adipose tissue inflammation. However, our knowledge of the effects of uremic toxins on adipose tissue remains limited at this time.

In this study, we focused on uremic toxins that are reported to accumulate in CKD patients, and found that such toxin is involved in adipose tissue inflammation. In an initial study, we searched for uremic toxin involved in adipocyte inflammation using 3T3-L1 cells that differentiate into adipocytes, and elucidated the molecular mechanism for the adipose tissue inflammation caused by uremic toxin. We then examined the contribution of uremic toxin in adipose tissue inflammation in vivo, using uremic toxin-overloaded mice. Finally, the effect of AST-120 on the adipose tissue inflammation in 5/6-nephrectomized CKD mice was also examined.

## 2. Results

### 2.1. Effect of Uremic Toxins on the Expression of the Monocyte Chemoattractant Protein-1 (MCP-1) and ROS Production in Adipocytes

**Figure 1:**
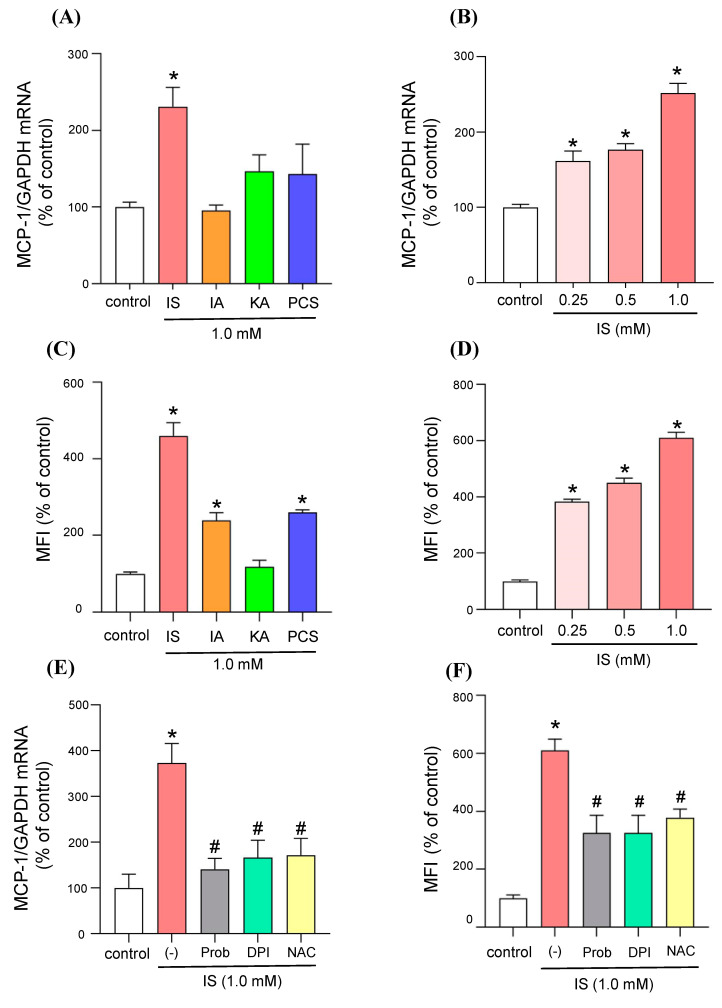
Effect of uremic toxins on monocyte chemoattractant protein-1 (MCP-1) expression and reactive oxygen species (ROS) production in adipocytes. (**A**) Differentiated 3T3-L1 adipocytes were incubated with 1 mM indoxyl sulfate (IS), 3-indoleacetic acid (IA), kynurenic acid (KA) or p-cresyl sulfate (PCS) for 24 h and MCP-1 mRNA expression was determined by quantitative RT-PCR. (**B**) Dose-dependency of IS-induced MCP-1 mRNA expression in differentiated 3T3L-1 adipocytes. (**C**) Differentiated 3T3-L1 adipocytes were incubated with 1 mM IS, IA, KA or PCS for 1 h to determine ROS production (shown as MFI: mean fluorescence intensity). (**D**) Dose-dependency of IS-induced ROS production in differentiated 3T3L-1 adipocytes. (**E**) Effect of organic anion transporter (OAT) inhibitors (probenecid (0.5 mM)), NADPH oxidase inhibitor (diphenylene iodonium: DPI (50  μM)) or antioxidant (N-acetyl-L-cysteine: NAC (0.5 mM)) on IS (1 mM)-induced MCP-1 expression (**E**) and ROS production (**F**) in differentiated 3T3-L1 adipocytes. Data are expressed as the mean ± SE. * *p* < 0.05 compared with control; # *p* < 0.05 compared with IS in the absence of inhibitor.

To examine the effect of uremic toxins on adipocyte inflammation, we compared the ability of each uremic toxin to induce MCP-1 using differentiated 3T3-L1 adipocytes. Four typical anionic protein-bound uremic toxins, indoxyl sulfate (IS), 3-indoleacetic acid (IA), p-cresyl sulfate (PCS) and kynurenic acid (KA), which are known to accumulate at high levels in CKD patients and are difficult to remove by dialysis were selected. Each uremic toxin (1 mM) was added to differentiated 3T3-L1 adipocytes and MCP-1 mRNA expression was measured by quantitative RT-PCR. The findings show that, among the four uremic toxins, a significant MCP-1 mRNA-inducing effect was observed only in the presence of IS (Figure 1A). The effect of IS on MCP-1 mRNA expression was concentration-dependent (0.25-1 mM), as observed in serum from CKD patients (Figure 1B) [15,16,17].

Next, in order to investigate the relationship between the induction of MCP-1 and the production of ROS, the production of ROS by each uremic toxin was examined using the differentiated 3T3-L1 adipocytes. The results indicated that a significant increase in ROS production occurred in the presence of IS, IA or PCS, and, among them, the highest ROS production was observed in the presence of IS (Figure 1C). In addition, IS was found to increase ROS production in a concentration-dependent manner (Figure 1D). These data suggest that ROS production by IS might contribute to the induction of MCP-1.

### 2.2. Molecular Mechanism of MCP-1 Induction by IS

IS is taken up by an organic anion transporter (OAT) in renal tubular cells, vascular endothelial cells, vascular smooth muscle cells and osteoblasts [18,19,20,21]. First, in order to investigate the pathway for MCP-1 induction by IS in adipocytes, the expression of OAT3 in adipocytes was evaluated by western blotting. As a result, similar to human renal proximal tubular epithelial cells (HK-2 cells), which were used as a positive control, a band was observed at 59 kDa, showing that OAT3 is expressed in differentiated 3T3-L1 adipocytes (Figure S1A). Probenecid, an OAT inhibitor, significantly suppressed the MCP-1 expression and ROS production induced by IS (Figure 1E,F). In addition, an NADPH oxidase inhibitor (diphenyleneiodonium chloride: DPI) or an antioxidant, N-acetylcysteine (NAC), significantly suppressed the IS-induced expression of MCP-1 and ROS production (Figure 1E,F). It was also found that IS increased the expression of NOX4, a subunit of NADPH oxidase, and that this effect was concentration-dependent (Supplementary Figure S1B), strongly suggesting that IS activates NADPH oxidase to produce ROS. These data indicate that IS is taken up by adipocytes via OAT, and that this leads to an increased production of intracellular ROS through the activation of NADPH oxidase, resulting in the induction of MCP-1 expression.

### 2.3. Effect of IS on Adipocytes and Macrophage Crosstalk

**Figure 2:**
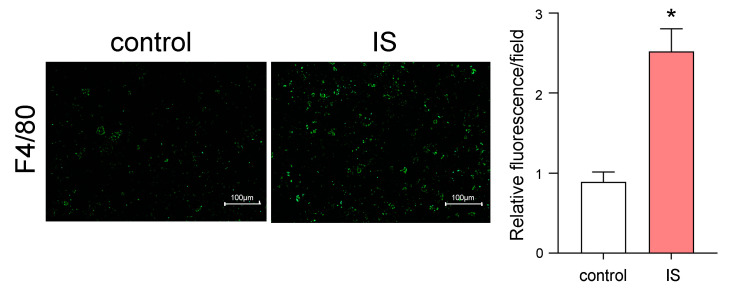
Measurement of macrophage recruitment to adipocytes by IS. Differentiated 3T3-L1 adipocytes and RAW264.7 macrophages were co-cultured in Transwell^®^ plates (differentiated adipocytes were in the lower side and RAW264.7 macrophages were in the upper side) in the presence or absence of IS (1 mM IS was added in the lower side) for 24 h. Infiltrated macrophages into the lower side were detected as F4/80 positive cells (green) using an anti-F4/80 monoclonal antibody. Original magnifications: ×200. Scale bars represent 100 μm. The F4/80-positive cells were quantitated using an imaging analysis program (BZ-X Analyzer, Keyence Inc., Osaka, Japan). Data are expressed as means ± SE. * *p* < 0.05 compared with control.

The effect of IS on adipocytes and macrophage crosstalk was evaluated by the co-culturing of adipocytes and macrophages using Transwell^®^ [22]. In this experiment, 3T3-L1 cells were seeded on the lower layer and differentiated to adipocytes and mouse macrophage cells (RAW264.7) were then seeded on the upper layer. At 24 h after the addition of IS to the adipocytes in the lower layer, macrophages that migrated from the upper layer to the lower layer were evaluated by fluorescent immunostaining using an anti-F4/80 antibody, a macrophage marker. As a result, a significant increase in the fluorescence intensity of F4/80 was observed in the presence of IS, compared to the absence of IS, suggesting the adipocytes with IS potentiate the increase in macrophage infiltration to adipocytes (Figure 2).

### 2.4. In Vivo Distribution of IS to Adipose Tissue and Its MCP-1 Inducing Effect: A Study Using IS-Overloaded Mice

**Figure 3:**
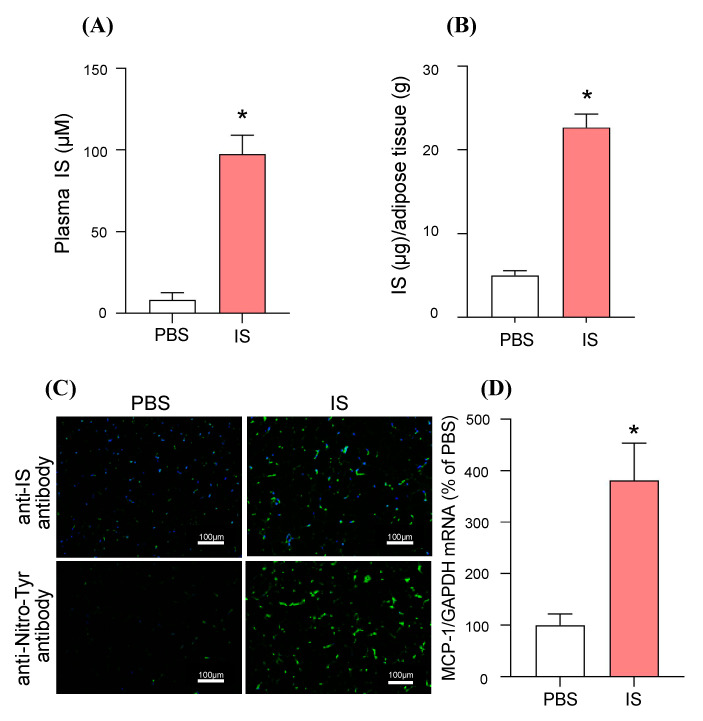
In vivo distribution of IS to adipose tissue and its MCP-1 inducing effect in IS-overloaded mice. The healthy mice were administrated with IS (100 mg/kg/day, ip). The control mice were administrated with the same volume of PBS. One hour after administration, the mice were anesthetized and blood, epididymal adipose tissue collected. IS levels in (**A**) plasma and (**B**) epididymal adipose tissue were measured by high-performance liquid chromatography (HPLC) methods. (**C**) IS accumulation (green) in epididymal adipose tissue was detected by immunofluorescence using anti-IS antibody. Immunofluorescent staining of nitrotyrosine (Nitro-Tyr: green) in epididymal adipose tissue was also shown. The section was also treated with DAPI (blue). Original magnifications: ×200. Scale bars represent 100 μm. (**D**) MCP-1 mRNA expression was determined by quantitative RT-PCR. Data are expressed as means ± SE (n = 4). * *p* < 0.05 compared with PBS-treated group.

The relationship between the distribution of IS and the expression of MCP-1 in adipose tissue was examined using IS-overloaded mice. The purpose of this experiment using IS-overloaded mice was to evaluate the in vivo distribution of IS to adipose tissue and its MCP-1 inducing effect, without affecting kidney function. In these experiments, IS (100 mg/kg) was intraperitoneally administered to healthy mice, and 60 min later, the IS levels in plasma and epididymal adipose tissue were measured by high-performance liquid chromatography (HPLC) [7]. Single intraperitoneally administration of IS did not affect the renal function (data not shown). As a result, the plasma IS levels in the IS-loaded mice were significantly increased (approximately 5-fold) compared to the PBS-treated group, and a significant increase in IS levels in epididymal adipose tissue was also observed in proportion to the plasma level (Figure 3A,B). Immunostaining using an anti-IS antibody in epididymal adipose tissue showed that an increase in fluorescence intensity was observed in the IS-loaded group compared to the PBS-treated group (Figure 3C). Then, oxidative stress marker, Nitro-Tyr in epididymal adipose tissue was also significantly increased in the IS-loaded group (Figure 3C). In the same experimental conditions, a significant increase in MCP-1 mRNA expression in epididymal adipose tissue was observed in the IS-loaded group as compared to the PBS-treated group (Figure 3D). These data confirm that IS could be distributed in adipose tissue and that it induces oxidative stress and MCP-1 expression in vivo.

### 2.5. Effect of AST-120 on Adipocyte Inflammation in 5/6-Nephrectomized CKD Mice

**Figure 4:**
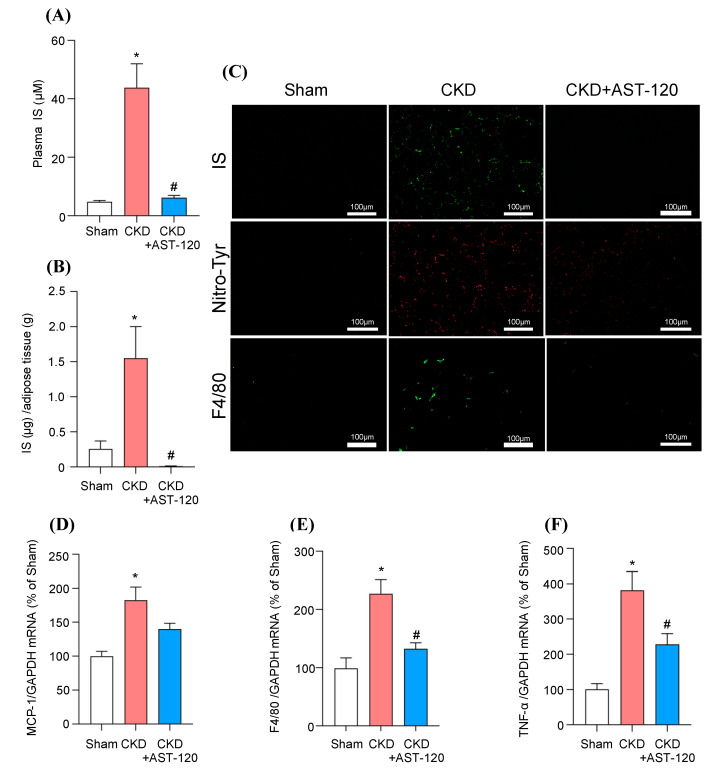
Effect of AST-120 on adipocyte inflammation in 5/6-nephrectomized CKD mice. After randomization at 4 weeks after nephrectomy, the AST-120-administered group was fed a diet containing 8% AST-120 for 24 weeks. Effect of AST-120 on IS levels in (**A**) plasma and (**B**) epididymal adipose tissue of 5/6-nephrectomized CKD mice. Immunofluorescent staining of (**C**) IS (green), nitrotyrosine (Nitro-Tyr: red)) and F4/80 (green) in epididymal adipose tissue of CKD mice. Original magnifications: ×200. Scale bars represent 100 μm. (**D**) MCP-1, (**E**) F4/80 and (**F**) TNF-α mRNA expression in epididymal adipose tissue were determined by quantitative RT-PCR. Data are expressed as means ± SE (n = 4). * *p* < 0.05 compared with sham; ^#^ *p* < 0.05 compared with CKD.

Finally, we investigated the effect of AST-120 on adipocyte inflammation in 5/6-nephrectomized CKD mice. AST-120 suppresses the absorption of indole, an IS precursor, from the intestinal tract, thereby lowering the IS levels in the body. As a CKD model, 5/6-nephrectomized mice were prepared and divided into three groups; (1) Sham group; (2) CKD group; and 3) CKD + AST-120 administration group, after randomization at 4 weeks after the nephrectomy. The AST-120-administered group was fed a diet containing 8% AST-120 for 24 weeks. At 28 weeks after the nephrectomy, no significant differences in blood urea nitrogen (BUN) and serum creatinine (SCr) were found between the CKD group and the CKD + AST-120 administration group (Table 1). The elevated IS levels in plasma and epididymal adipose tissue in CKD mice were significantly suppressed by the administration of AST-120 (Figure 4A,B). Regarding the distribution of IS in epididymal adipose tissue, similar results were obtained by immunostaining using an anti-IS antibody (Figure 4C).

Oxidative stress (Nitro-Tyr) (Figure 4C), MCP-1 (Figure 4D) and F4/80 (Figure 4C,E) in epididymal adipose tissue were significantly increased in the CKD mice, and these increases were suppressed by the administration of AST-120 (Figure 4C–E). The expression of the proinflammatory adipocytokine TNF-α mRNA in epididymal adipose tissue was significantly increased in the CKD mice, but AST-120 administration suppressed this effect (Figure 4F). These results are consistent with the above in vitro data, and suggest that IS contributes to adipose tissue inflammation in CKD pathogenesis. In addition, we also found that AST-120 was effective in reducing CKD-induced adipose tissue inflammation.

## 3. Discussion

In the present study, we found that IS distributed to adipose tissue and then induces MCP-1 expression via the OAT/NADPH oxidase/ROS pathway. IS-induced MCP-1 expression could allow macrophages to infiltrate into adipocytes, resulting in an increase in adipose tissue inflammation. These data suggest that IS primes adipocytes for an inflammatory response. This conclusion is strongly supported by the finding that the adipose tissue inflammation caused by IS in CKD mice was reduced by AST-120 administration.

Among some uremic toxins, such as IS, IA, KA and PCS, ROS production and MCP-1 induction were highest in adipocytes that were incubated with IS (Figure 1A,C). There are two possible reasons for the differences in ROS production and MCP-1 expression that were observed in the presence of each uremic toxin. The first is the differences in intracellular uptake via OAT. OAT, an intracellular uptake transporter, has a higher affinity for IS than for IA, KA or PCS [12,23,24]. In fact, our data showed that OAT was also expressed in differentiated 3T3-L1 adipocytes (Figure S1A), and probenecid, an OAT inhibitor, inhibited IS-induced ROS production and MCP-1 expression (Figure 1E,F). Based on these results, it appears that the mechanism responsible for the intracellular uptake of IS via OAT is the first step in the action of IS, and that the differences in the substrate specificity of each uremic toxin by OAT may contribute to their action on adipose tissue. Although IA and PCS also significantly increased ROS production (Figure 1C), they did not induce MCP-1 expression (Figure 1A), unlike IS in 3T3-L1 adipocyte. These data suggested that the amount of ROS production in 3T3-L1 adipocyte could be important to induce MCP-1 expression.

The second reason is the possible involvement of an aryl hydrocarbon receptor (AhR) in the action of IS. We previously reported that IS induces muscular atrophy through AhR in muscle cells (C2C12 cell) [7], and there have been many reports that IS is an endogenous ligand for AhR [25,26,27]. Therefore, the involvement of AhR in the action of IS in 3T3-L1 adipocytes was examined using an AhR inhibitor (CH223191). Unexpectedly, CH223191 did not significantly suppress the IS-induced production of ROS and MCP-1 expression (data not shown). In a previous study, Hamano et al. reported that IS produces AhR-independent ROS in hepatocytes (HepG2 cells) [28]. Bartlett et al. also demonstrated that Na/K-ATPase in adipocytes amplifies IS-derived ROS production [29]. Our data show that IS increased the expression of NOX4 (Figure S1B), leading to the activation of NADPH oxidase. Taking these previous reports and our findings into consideration, the possibility that IS activates NADPH oxidase in an AhR-independent manner to produce ROS in adipocytes cannot be excluded.

Han et al. reported that NOX4 is the most highly expressed NOX isoform in cultured murine and human adipocytes [30]. Other NOX family members were detected at very low levels. They also found that, in adipocytes, excess glucose and palmitic acid could induce NOX4-derived ROS production [30]. Furthermore, De Hartigh et al. tested a high fat diet-load on adipocyte-specific NOX4-deficient mice, and found that NOX4-derived ROS in adipocytes is directly involved in the development of insulin resistance and adipose tissue inflammation, suggesting that NOX4-derived ROS is involved in obesity-related adipose tissue inflammation [31]. The enhancement in NOX4 expression by IS (Figure S1B) suggests that IS, glucose and palmitate are involved in adipose tissue inflammation through a common NOX4-ROS pathway. In the future, the combination effects of glucose, palmitate and IS on adipose tissue inflammation may need to be further clarified.

The ASK-1/NF-κB/MAPKs pathway may be involved as a pathway for IS-induced MCP-1 expression. In tubular cells and cardiomyocytes, intracellular ASK-1/thioredoxin complex senses IS-induced ROS, which promotes phosphorylation of NF-κB and MAPKs followed by the expression of inflammatory cytokines [32]. In adipocytes, it was also reported that the expression of inflammatory cytokines, including MCP-1, were increased through the activation of NF-κB and MAPKs as a downstream of Na/K-ATPase activation [29]. These collective findings suggest that IS-derived ROS induces MCP-1 expression through the activation of ASK-1/NF-κB/MAPKs and Na/K-ATPase in adipocytes. Further study will be clearly needed to confirm the contribution of these signaling pathway in the future.

The co-culture study of differentiated 3T3-L1 adipocytes and RAW264.7 mouse macrophage cells using Transwell^®^ showed that IS increased the infiltration of macrophages into adipocytes. Our in vitro study using differentiated 3T3-L1 adipocytes showed that IS increases the expression of MCP-1 in adipocytes, which can stimulate the infiltration of macrophages. These data suggest that IS acts on adipocytes and induces inflammation by enhancing the migration of surrounding monocytes and macrophages into adipose tissue. In fact, in the IS-loaded mice, an increase in IS accumulation and MCP-1 expression in adipose tissue were observed (Figure 3).

The administration of AST-120 to 5/6-nephrectomized CKD mice suppressed the accumulation of IS in adipose tissue (Figure 4B). Sato et al. recently reported that the administration of AST-120 to adenine-induced CKD mice did not significantly suppress the accumulation of IS in adipose tissue [33]. This difference may be due to the long-term (24 weeks) administration of AST-120 in our study, whereas the feeding period of AST-120 was 4 weeks in their study [33]. That is, it would be expected that the long-term administration of AST-120 could suppress the accumulation of IS in adipose tissue. As shown in Figure 4, in the adipose tissue of CKD mice, an increase in MCP-1 expression and F4/80 expression was observed, indicating that macrophage infiltration into adipose tissue had occurred. In addition, TNF-α expression was also increased in adipose tissue, suggesting the occurrence of an inflammatory response in adipose tissue in the CKD mice. These data are in general agreement with previous reports in which CKD rats were used [3]. In our experimental conditions, although AST-120 administration did not significantly alleviate the renal function of CKD mice, the adipose tissue inflammation was suppressed by AST-120. These data support the conclusion that IS induces adipose tissue inflammation under CKD conditions.

## 4. Conclusions

The findings reported in this study show the IS could be an endogenous factor that induces adipose tissue inflammation. We also demonstrated that the administration of AST-120 is useful for reducing IS-induced adipose tissue inflammation. Taken together, IS could be a therapeutic target for the CKD-related inflammation of adipose tissue.

## 5. Materials and Methods

### 5.1. Chemicals and Materials

IS, 3-indoleacetic acid, kynurenic acid, probenecid, diphenylene iodonium (DPI), insulin, dexamethasone, isobutylmethylxanthine, were purchased from Sigma-Aldrich (St Louis, MO, USA). PCS was synthesized according to a previous report [34]. The rabbit anti-OAT3 polyclonal antibody (bs-0609R) was purchased from Bioss Inc (Boston, MA, USA). The anti-IS antibody was kindly provided by the Kureha Corporation (Tokyo, Japan). The anti-F4/80 monoclonal antibody was purchased from eBioscience (San Diego, CA, USA). The nitro-Tyr antibody was purchased from Millipore (Burlington, MA, USA). N-acetyl-L-cysteine was purchased from nacalai tesque (Kyoto, Japan). Dulbecco’s phosphate-buffered saline (D-PBS), 5-(and 6)-chloromethyl-2,7’-dichlorodihydrofluorescein diacetate (CM-H2DCFDA) was purchased from Gibco (Invitrogen, Grand Island, NY, USA). Dulbecco’s modified eagle medium (DMEM)-high glucose and DMEM-low glucose was purchased from Wako (Osaka, Japan). Penicillin/streptomycin (Thermo Fisher Scientific, Waltham, MA, USA). All procedures were carried out in accordance with approved guidelines. All experimental protocols were approved by Kumamoto University.

### 5.2. Cell Cultures

Mouse 3T3-L1 fibroblasts were cultured in DMEM-low glucose supplemented with 10% (*v*/*v*) bovine calf serum (GE Healthcare, UK Ltd., Backinghamshire, UK) and 1% penicillin/streptomycin. Differentiation into adipocytes was induced by exposing post-confluent cells for 2 days to induction medium. The induction medium consisted of DMEM-high glucose supplemented with 10% FBS (CORNING), 1% penicillin/streptomycin, 10 μg/mL insulin, 2.5 μM dexamethasone, 0.5 mM isobutylmethylxanthine. After two days, the media was changed to maturation medium. The maturation medium consisted of DMEM-high glucose supplemented with 10% FBS, 1% penicillin/streptomycin, 10 μg/mL insulin. The mature media were exchanged every other day until day 12. The cells that have been subjected to the above treatment were differentiated adipocytes (Figure S2). RAW264.7 cells (Mouse Mφ cells) were grown in DMEM-high glucose supplemented with 10% FBS, 1% penicillin/streptomycin at 37 °C in a humidity incubator within 5% CO₂.

### 5.3. Quantitative RT-PCR

The reverse transcription of total RNA from differentiated adipocytes and adipose tissue by the RNAiso plus (Takara Bio) was performed using a PrimeScript RT Master Mix (Takara Bio Tokyo, Japan). Quantitative RT-PCR was performed under an iCycler thermal cycler (Bio-Rad Laboratories, Hercules, CA, USA) using SYBR Premix Ex TaqII (Takara Bio). Performed by quantitative RT-PCR, as described in a previous report [8], and the primers used are listed in the Table S1. GAPDH was used as an internal control.

### 5.4. ROS Production Assay

The ROS production assay was performed using CM-H2DCFDA, which changes into DCF by the ROS generated in cells and emits a fluorescence. 3T3-L1 cells were seeded in a 96-well plate and allowed to differentiate into adipocytes. After cell differentiation, the cells were washed with PBS and starved with serum free medium for 2 h and then treated with CM-H2DCFDA (5 μM) for 30 min in D-PBS. After removing the D-PBS, the cells were incubated with uremic toxins (IS, PCS, IA, KA) in D-PBS for 1 h. To determine the effect of inhibitors of OATs, NADPH oxidase, antioxidants on the IS-induced ROS production, cells were incubated with CM-H2DCFDA for 30 min in D-PBS. After removing the D-PBS, the cells were incubated with OATs inhibitors (probenecid (0.5  mM)), NADPH oxidase inhibitor (diphenylene iodonium: DPI (50  μM)), antioxidant (N-acetyl-L-cysteine (0.5 mM)) for 30  min, and then incubated with IS (1  mM) in D-PBS for 1 h. Fluorescence intensity (excitation wavelength: 485 nm, fluorescence wavelength: 535 nm) was measured by a fluorescence microplate reader SPECTRAfluor Plus (Tecan, Männedorf, Switzerland).

### 5.5. Measurement of the Recruitment of Macrophages to Adipocytes

The 3T3-L1 cells were differentiated into adipocytes on glass coverslips. The differentiated adipocytes and RAW264.7 macrophages were co-cultured in Transwell plates (differentiated adipocytes in the lower side and RAW264.7 macrophage in the upper side; pore size, 4 μm) in the presence or absence of IS (1 mM: IS was added in the lower side) for 24 h. After discarding the upper plates, the cells on the coverslips (lower side) were washed three times with PBS, fixed with 3.7% paraformaldehyde, permeabilized by treatment with PBS in 0.5% Triton X-100 (PBS-T), incubated with 0.1% PBS-T containing 3% bovine serum albumin for blocking for 1 h, and subsequently incubated with F4/80 monoclonal antibody at room temperature for 1 h and washed with 0.1% PBS-T. The cells were then incubated at room temperature with FITC-conjugated secondary antibodies. The coverslips were rinsed and placed on a glass slide with a mounting solution containing 4’,6-diamino-2-phenylindole. The cells were then visualized using a fluorescence microscope (BZ-X700: Keyence Inc. Japan), and the F4/80-positive cells were quantitated using an imaging analysis program (BZ-X Analyzer).

### 5.6. Animal Experiments

All animal experiments were carried out in accordance with approved the guidelines of Kumamoto University for the care and use of laboratory animals. All animal experiments were conducted using procedures approved by the experimental animal ethics committee at Kumamoto University (the project identification code, A2019-017; date of approval, 03 October, 2019). ICR mice (5 weeks, male) were purchased from Japan SLC (Shizuoka, Japan) and bred on a 12 h day/night cycle. Mice were administrated with IS (100 mg/kg/day, ip). The control mice were administrated with PBS at the same volume. At 1 h after administration, the mice were anesthetized with diethyl ether and blood, epididymal adipose tissue were collected.

The 5/6-nephrectomized model mice were produced in a two-step surgery procedure, according to previous reports [8]. In summary, two-thirds of the right kidney was removed, and 1 week later, the left kidney was completely removed. At 4 weeks after the final surgery, the mice were randomized by blood urea nitrogen (BUN) and body weight. The mice were assigned to AST-120 (charcoal oral absorbent, 8 w/w% in powder diet) treatment groups. Sham-operated mice and control mice (CKD-operated mice) received a normal diet and water. At 24 weeks after the final surgery, the mice were sacrificed, and blood, epididymal adipose tissue collected.

### 5.7. High-Performance Liquid Chromatography (HPLC) Analysis

IS levels in the plasma and adipose tissue were measured by an HPLC method, as described previously [7]. In brief, plasma or a adipose tissue homogenate extracted with RIPA buffer containing 150 mM NaCl, 1% nonidet P-40, 10 mM Tris-HCl (pH 7.4), and 1% protease inhibitor cocktail (Nacalai Tesque, Kyoto, Japan) was mixed with acetonitrile (1:9, *v*/*v* for the plasma sample or 1:3, *v*/*v* for the tissue homogenate sample) and centrifuged at 12,000× *g* for 10 min. The supernatant was collected and mixed with ultrapure water (1:1, *v*/*v* for a plasma sample or 3:2, *v*/*v* for a tissue homogenate sample). The sample was loaded to the HPLC with 20 μL being used for the plasma sample and 50 μL for the tissue homogenate. The HPLC system consisted of an Agilent 1100 series intelligent pump and a fluorescence spectrophotometer. A Shiseido (Tokyo, Japan) CAPCELL PAK C18 column (150 × 2.0 mm, 5 μm) was used as the stationary phase. The mobile phase consisted of 0.2 M acetate buffer (pH 4.0)-acetonitrile (3:1, *v*/*v*) for IS. The flow rate was 1.0 mL/min. Indoxyl sulfate was detected by means of a fluorescence monitor, with excitation/emission wavelengths set to 280 nm and 375 nm, respectively.

### 5.8. Immunostaining Assay

Epididymal adipose tissue, were fixed in 4% paraformaldehyde and embedded in paraffin or Tissue-Tek OCT medium. The deparaffinized sections were antigen-activated with Histo VT One (Nacalai Tesque, Kyoto, Japan) and incubated with primary antibody overnight at 4 °C. The IS antibody, Nitro-Tyr antibody, and F4/80 antibody were selected as primary antibodies, followed by incubation with the secondary antibody (Alexa 488 donkey anti-goat IgG(H + L) (Invitrogen, Waltham, MA, USA) or Alexa647 Goat Anti-Rat IgG (H + L) Antibodies (Abcam plc, Cambridge, UK) or Alexa488 Goat Anti-Rat IgG (H + L) Antibodies (Abcam plc, Cambridge, UK)) for 90 min at room temperature. Images were obtained on a Keyence BZ-X710 microscope (Keyence, Osaka, Japan). The average fluorescence intensity of the epididymal fat sections was quantified using an image analysis program (BZ-X analyzer: Keyence, Osaka, Japan).

### 5.9. Western Blotting

Total protein was extracted by using RIPA buffer containing 150  mM NaCl, 1% nonidet P-40, 10  mM Tris-HCl (pH7.4), 1% protease inhibitor cocktail (nacalai tesque, Kyoto, Japan). A 10 μg sample of protein was mixed with sample buffer containing 50 mM dithiothreitol, boiled at 100 °C, and then separated with 10% sodium dodecyl sulfate-poly-acrylamide gel electrophoresis. Proteins were transferred polyvinylidene fluoride membrane, and then immunoblotted with antibodies against mouse OAT3, β-actin under the 4 °C for 16 h. The sample was then immunoblotted with horseradish peroxidase conjugated secondary antibody at room temperature for 1 h. The intensity of each band was detected using LAS4000mini (GE Healthcare, UK Ltd., Backinghamshire, England).

### 5.10. Biochemical Evaluation in Plasma

BUN and serum creatinine were measured using FUJI DRI-CHEM (Fujifilm, Tokyo, Japan). The values for the biochemical evaluation in plasma from CKD mice are listed in Table 1.

### 5.11. Statistical Analyses

Since no continuous variables were normally distributed according to the frequency distribution and Q-Q plot, the Mann-Whitney U test was used for comparisons. A value of *p* < 0.05 was considered to be statistically significant. Multiple comparisons were corrected using Bonferroni’s method, and values of *p* < 0.05/n were considered to be statistically significant after correcting the number of comparisons made in the Statistical Analysis of the revised manuscript.

## Figures and Tables

**Table 1:** Plasma biochemical parameters in chronic kidney disease (CKD) mice. Renal function for sham, 5/6-nephrectomized (CKD) and AST-120 treated CKD mice.

	Sham	CKD	CKD + AST-120
BUN (mg/dL)	21.1 ± 1.2	56.7 ± 9.4 ^a^	45.5 ± 6.0 ^a^
SCr (mg/dL)	0.2 ± 0.02	0.8 ± 0.31 ^a^	0.4 ± 0.13

Data are expressed as the mean ± SE (n = 4). ^a^ *p* < 0.05 compared with sham. BUN, blood urea nitrogen; SCr, serum creatinine.

## Supplementary Materials

The following are available online at https://www.mdpi.com/2072-6651/12/8/502/s1, Figure S1: (A) OAT expression in differentiated 3T3-L1 adipocytes. Human renal proximal tubular epithelial cells (HK-2 cells) were used as a positive control, a band was confirmed at 59 kDa verifying the presence of OAT3 in differentiated 3T3-L1 adipocytes. (B) Effect of IS on NOX4 mRNA expression in adipocytes. IS increased NOX4 mRNA expression in a dose-dependent manner. Figure S2. Intracellular lipid droplets were stained with Oil Red O on day 14 of 3T3-L1 adipocyte differentiation and observed by microscopy. Table S1: Primers used in real-time RT-PCR.
