# Supplementary material for: Indoxyl Sulfate Contributes to Adipose Tissue Inflammation through the Activation of NADPH Oxidase
Source: Toxins (Basel). 2020 Aug 5;12(8):502. doi: 10.3390/toxins12080502 (PMC7472142; doi:10.3390/toxins12080502)
Supplement: Supplementary file 1 [file toxins-12-00502-s001.pdf]

Supplementary Materials: Indoxyl Sulfate Contributes to Adipose Tissue Inflammation through the Activation of NADPH Oxidase

Shoma Tanaka, Hiroshi Watanabe, Takehiro Nakano, Tadashi Imafuku, Hiromasa Kato, Kai Tokumaru, Nanaka Arimura, Yuki Enoki, Hitoshi Maeda, Motoko Tanaka, Kazutaka Matsushita, Masafumi Fukagawa and Toru Maruyama

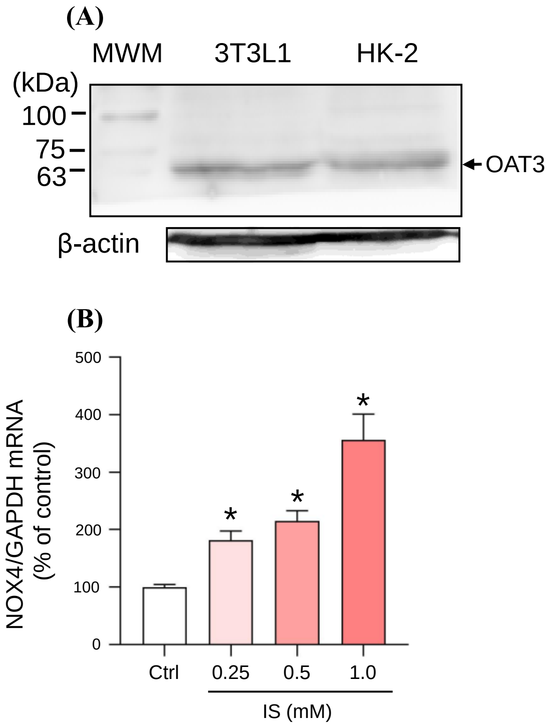

Figure S1. (**A**) OAT expression in differentiated 3T3-L1 adipocytes. Human renal proximal tubular epithelial cells (HK-2 cells) were used as a positive control, a band was confirmed at 59 kDa verifying the presence of OAT3 in differentiated 3T3-L1 adipocytes. (**B**) Effect of IS on NOX4 mRNA expression in adipocytes. IS increased NOX4 mRNA expression in a dose-dependent manner. Data are expressed as the mean ±SE. $^{*}p < 0.05$, $^{**}p < 0.01$ compared with control.

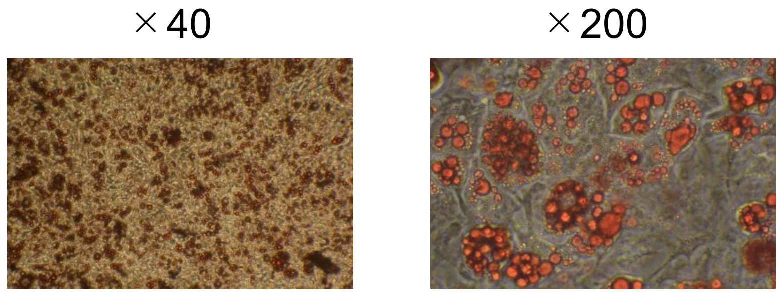

Figure S2. Intracellular lipid droplets were stained with Oil Red O on day 14 of 3T3-L1 adipocyte differentiation and observed by microscopy (original magnification ×40 and ×200).

Table S1. Primers used in real-time RT-PCR.

Target Gene	Forward	Reverse
MCP-1	5′-TGCATCTGCCCTAAGGTCTTC-3′	5′-AAGTGCTTGAGGTGGTTGTGG-3′
F4/80	5′-CATAAGCTGGGCAAGTGGTA-3′	5′-GGATGTACAGATGGGGGATG-3′
TNF-α	5′-CATGAGCACAGAAAGCATGATCCG-3′	5′-AAGCAGGAATGAGAAGAGGCTGAG-3′
Nox4	5′-ATTTGGATAGGCTCCAGGCAAAC-3′	5′-CACATGGGTATAAGCTTTGTGAGCA-3′
GAPDH	5′-AACTTTGGCATTGTGGAAGG-3′	5′-ACACATTGGGGGTAGGAACA-3′
